# Balloon Occlusion Types in the Treatment of Coronary Perforation during Percutaneous Coronary Intervention

**DOI:** 10.1155/2014/784018

**Published:** 2014-11-20

**Authors:** Xiangfei Wang, Junbo Ge

**Affiliations:** Department of Cardiology, Shanghai Institute of Cardiovascular Diseases, Zhongshan Hospital, Fudan University, 180 Fenglin Road, Shanghai 200032, China

## Abstract

Coronary artery perforation is an uncommon complication in patients with coronary heart disease undergoing percutaneous coronary intervention. However, pericardial tamponade following coronary artery perforation may be lethal, and prompt treatment is crucial in managing such patients. Balloon occlusion and the reversal of anticoagulant activity are the common methods used to prevent cardiac tamponade by reducing the amount of bleeding. Herein, we discuss the pros and cons of currently used occlusion types for coronary perforation. Optimal balloon occlusion methods should reduce the amount of bleeding and ameliorate subsequent myocardial ischemia injury, even during cardiac surgery.

## 1. Introduction

Coronary artery perforation (CAP) is an uncommon but potentially lethal complication in patients with coronary heart disease who are undergoing percutaneous coronary intervention (PCI). The first large-scale clinical investigation of the incidence of CAP associated with PCI was conducted by Ellis et al. [1]. Their two-year registry included 12,900 cases of PCI. The incidence of CAP was 0.5% and was more common in elderly female patients. CAP was classified according to the Ellis scheme. Type I CAP is defined by the development of an extraluminal crater, without extravasation. Type II CAP is defined by the development of a pericardial or myocardial blush, without contrast jet extravasation. Type III CAP is defined by the development of an extravasation jet through a frank (≥1 mm) perforation or cavity spilling into an anatomic cavity chamber (ventricles, pericardial space, etc.). The incidence of pericardial tamponade in type I CAP patients was 8%, and there have been no reports of mortality. A total of 63% of patients with type III CAP developed pericardial tamponade, and the mortality rate was 19%. Subsequently, a meta-analysis of 16 studies (197,061 cases) reported that the incidence of CAP was 0.43% (95% CI, 0.35%–0.52%) [2]. The most valuable predictors are disease complexity and utilization of plaque ablation. The incidence and mortality of pericardial tamponade were closely associated with the Ellis classification (e.g., type I, 0.4% and 0.3%; type II, 3.3% and 0.4%; type III, 45.7% and 21.2%, resp.). Moreover, type III CAP is associated with high long-term major adverse cardiac event rates [3].

There are a variety of management methods for CAP, including observation, heparin reversal [4], prolonged balloon occlusion [5], membrane-covered stent placement [6], embolization [7], pericardiocentesis [8], and surgical treatment [9]. Prolonged balloon occlusion and reversing the anticoagulant activity of heparin are the most commonly used methods. Prolonged balloon occlusion has proven effective, and the occlusion time varies from 5 to 15 min [10]. Unfortunately, there is little chance of success by single balloon occlusion. Longer occlusion times and repeated occlusion are common situations [11]. However, a prolonged total occlusion time may induce myocardial dysfunction or cardiomyocyte necrosis. In this review, we discussed various balloon occlusion models and attempt to identify the optimal model that can both reduce the amount of bleeding and ameliorate subsequent myocardial ischemia injury, even during cardiac surgery. A detailed discussion of CAP can be found in other publications [12–14].

## 2. Occlusion Types

Based on the duration and completeness of the occlusion, there are four balloon occlusion models: persistent complete occlusion, persistent partial occlusion, intermittent complete occlusion, and intermittent partial occlusion.

### 2.1. Persistent Complete Occlusion

When coronary artery blood flow is completely blocked, myocardial necrosis exhibits a wavefront phenomenon in which the myocardial necrosis spreads from the subendocardium to the subepicardium. Similar to the human coronary circulation, the porcine coronary collateral circulation is sparse. One study of porcine hearts reported no significant myocardial necrosis after 20 min of coronary occlusion but became significant after 30 min of coronary occlusion; after 60 min of coronary occlusion, most cardiomyocytes were necrotic [15]. Large animals are usually more tolerant to ischemia than small animals. Therefore, more than 20 min of complete coronary occlusion is unacceptable for humans. However, in such a short period, the thrombus may not be sufficiently stable to stop bleeding at the location of the perforation. An additional disadvantage of persistent complete occlusion is that the anticoagulant effect of heparin cannot be reversed by protamine because protamine cannot reach the perforation place secondary to stagnant blood flow. Thus, persistent complete occlusion is not an ideal solution.

### 2.2. Persistent Partial Occlusion

To our knowledge, there have been no reports on whether severe artificial stenosis caused by persistent partial occlusion can be used to manage CAP. However, this method is theoretically possible. During severe artificial stenosis, the pressure at the distal coronary artery stenosis is significantly reduced and the rate of bleeding from the perforation site subsequently decreases. Pericardial tamponade can be avoided if the amount of bleeding is remarkably reduced below the upper limit of the pericardial reserve volume. A better understanding of the relationship between the degree of coronary artery stenosis and the amount of CAP bleeding will benefit the use of artificial stenosis in reducing bleeding.

#### 2.2.1. Amount of Bleeding and Blood Pressure Reduction

Controlled hypotension is routinely used to reduce the amount of bleeding during surgical procedures. For nonhypertensive patients, the mean arterial pressure (MAP) should be maintained at 50–65 mmHg. For hypertensive patients, MAP should be controlled at 30% below baseline. Most studies have shown that this method reduces <40% of bleeding [16]. Because the capacity of the pericardial cavity is extremely limited, the local arterial pressure should be further reduced as much as possible to avoid cardiac tamponade in patients with CAP. Ryba et al. attempted to reduce MAP from 90 mmHg to 35 mmHg during cerebral aneurysm operations, and no obvious evidence of ischemia was found [17]. Therefore, it is reasonable to assume that a distal MAP range from 35 to 50 mmHg is likely to be an optimal target for CAP management.

#### 2.2.2. Reduction of Blood Pressure and Fractional Flow Reserve (FFR)

By controlling the degree of stenosis of the circumflex artery in canines, the myocardial contractile thickness was reduced to 50–75% of normal for 5 hrs. During the artificial ischemic process, the subendocardial blood flow was decreased by 55%. However, after the myocardial blood supply was restored, myocardial contractility was not completely restored to normal for 7 days [18]. Histological examination revealed only mild damage to cardiomyocytes in the ischemic area. This phenomenon is known as “myocardial stunning.” The myocardium recovery time increases with the severity of the ischemia. Schulz et al. used a similar method to further determine blood pressure after porcine coronary artery artificial stenosis [19]. In the control group, the mean coronary artery pressure was 111 mmHg, while, in the stenosis group, the mean coronary artery pressure of the distal stenosis was maintained at approximately 40 mmHg, and the blood flow was reduced to 40% of normal. Thus, it appears that maintaining the distal MAP at 40 mmHg for a short period can prevent cardiomyocyte necrosis. According to the theory of FFR measurements, the resting coronary FFR (FFRcor) in this artificial stenosis is approximately 0.33 and the pressure difference across the stenosis site (Δ*P*) is approximately 70 mmHg.

#### 2.2.3. FFR and Coronary Artery Stenosis

In the resting state, the local myocardial blood supply was not reduced until the severity of the coronary artery stenosis (measured in diameter) reached >80% [20]. When the artificial stenosis reaches approximately 80%, Δ*P* is >60 mmHg by analyzing the monitoring curve [21]. Bartúnek et al. used a pressure guide wire to directly determine the Δ*P* of 110 patients with normal cardiac function who underwent nonselective coronary interventions [22]. FFRcor was well correlated with the myocardial FFR (FFRmyo), and FFRmyo was approximately 0.5 when FFRcor was 0.4. The severity of the stenosis and minimum lumen diameter was curvilinear related: when FFRmyo was 0.5, the severity of stenosis was approximately 70%, and the minimum lumen diameter was approximately 1.0 mm; when FFRmyo was 0.4, the severity of stenosis was 80%, and the minimum lumen diameter was 0.5 mm. Thus, a minimum lumen diameter between 0.5 and 1.0 mm may be the acceptable target. In the FIRST study, both pressure guide wire and intravascular ultrasound were used to investigate the relationship between the FFR and the minimum luminal cross-sectional area [23]. Only a moderate correlation between the two measurements was found, and the diameter of the reference vessel significantly impacted the correlation between the two measurements. Therefore, the diameter of the proximal reference vessel should also be considered when artificial stenosis is applied.

Interestingly, low perfusion resulting from persistent partial occlusion can improve the tolerance of the heart to reperfusion injury in pigs, a process that is similar to ischemic preconditioning [24]. The infarct size was decreased in the group with 70% reduction of coronary blood flow whereas no significant change occurred in the group with 30% reduction.

In summary, an optimal distal MAP in the persistent partial occlusion model should be maintained at approximately 40 mmHg in the resting state, with Δ*P* > 60 mmHg and FFRcor < 0.4. According to these criteria and considering individual variation, ≥90% stenosis should be achieved by the partial occlusion. A short-length, high-compliance balloon should be inflated at the location of CAP, and the balloon diameter can be adjusted as necessary by the pressure. We speculate that thrombolysis in myocardial infarction (TIMI) flow grading might also be a valuable monitoring parameter, with TIMI flow grade 2 as the potential goal during the partial occlusion. However, there are no related studies. In addition, along with the increased pericardial pressure caused by the accumulation of blood in the pericardial cavity due to ongoing bleeding, local myocardial perfusion pressure is also gradually decreased. To prevent this excessive low perfusion pressure of the local myocardial tissue, pericardiocentesis can be adopted to reduce the pericardial pressure to restore the minimum local perfusion status.

### 2.3. Intermittent Complete Occlusion

As mentioned above, the most commonly used method is prolonged balloon occlusion which can be regarded as an intermittent complete occlusion model according to our classification. An intermittent, nonfatal complete ischemic model had been found to improve tissue tolerability to reperfusion injury after long-term ischemia and to delay cell death [25]. This phenomenon is known as “ischemic preconditioning (IPC).” The classic model to activate the preconditioning effect is four cycles of 5 min occlusion followed by 5 min of reperfusion. Compared with classic IPC, however, a longer ischemia time and shorter reperfusion time in this type of balloon occlusion are required to minimize bleeding in CAP on the condition that the beneficial effects of IPC persist. A single coronary ischemia that lasts for <2 min may be insufficient to activate IPC. Many studies have suggested that a 10 min ischemia time is an ideal time setting to induce IPC [26, 27]. Because the preconditioning effect can be quickly saturated, further stimulation will not produce additional effects. The requirement for the reperfusion time is not as stringent as for the ischemia time. In rat hearts, a 30 s reperfusion time was insufficient, but a 60 s reperfusion is sufficient to successfully activate preconditioning effect [28]. To date, there have been no related human studies.

Coronary heart disease patients are often exposed to one or more cardiovascular disease risk factors, including (but not limited to) smoking, hypertension, diabetes, hypercholesterolemia, and advanced age. The impact of cardiovascular disease risk factors on IPC is still unknown. The effect of IPC may be weakened or even blocked by these factors; however, it can be partially restored by increasing the stimulation of IPC [29]. Another factor that may influence the IPC effects is stenosis itself. Kapadia et al. found that the infarct sizes in the IPC group and in the stenosis combined with IPC group were significantly reduced compared with the control group; however, in the simple stenosis group, the infarct size was not reduced. This result indicates that severe stenosis did not activate or prevent the activation of preconditioning [30].

The protection of IPC can be divided into two time phases: early and late. The early IPC phase occurs within the first 2-3 hrs after an ischemic event, whereas the late phase occurs between 12–24 hrs and 48–72 hrs. If cardiac surgery is unavoidable for patients with pericardial tamponade, the best time window is within 3 hrs after balloon occlusion, when the protective effect of IPC is maximal.

IPC can protect myocardial tissue in both ischemic and nonischemic areas. The protection for nonischemic myocardial tissue is called remote IPC, which was first described by Przyklenk et al. [31]. Subsequently, this phenomenon was found to exist widely in a variety of tissues and organs of different animals. Many small-scale clinical studies have found that remote IPC through limb ischemic stimulation can reduce the severity of myocardial infarction in patients with acute myocardial infarction [32] and reduce the incidence of contrast agent-induced nephropathy in patients undergoing elective coronary angiography [33]. Furthermore, it has been proven that remote IPC is also safe in patients with subarachnoid hemorrhage [34]. Cardiac preconditioning, which is a remote IPC for other vital organs, can provide comprehensive protection during emergency cardiac surgery for CAP, especially in patients with chronic kidney disease.

We speculate that a 10 min ischemia/1 min reperfusion model is optimal. If necessary, this procedure can be repeated three or four times. Notably, IPC can attenuate ischemia reperfusion injury during cardiac surgery, which is the ultimate method to treat CAP [35]. However, this model may not be satisfactory for patients with preexisting cardiac dysfunction. To avoid cardiac function deterioration, a shortened ischemic time or prolonged reperfusion time (such as 5 min/5 min) model should be an alternative in these patients.

### 2.4. Intermittent Partial Occlusion

To verify whether a CAP has been sealed during partial occlusion, a balloon must be deflated for angiography, which results in reperfusion. If the perforation is not sealed, further occlusion is needed (i.e., the intermittent partial occlusion model). This process can also induce ischemia reperfusion injury, in which the ischemic myocardium shifts from stunned to hibernating. Because of the heavy total ischemic burden, hibernating cardiomyocytes are damaged more severely than stunned cardiomyocytes are; therefore, the remarkably prolonged recovery time is usually a few days to several months [36].

## 3. Neutralization of Anticoagulant Activity

To prevent thrombotic events during PCI, anticoagulants are routinely used. Therefore, in cases of CAP bleeding, local thrombus formation and stabilization require a longer period than normal. Even if the slow or stagnant blood flow from the balloon occlusion is beneficial to thrombosis, the possibility of stable thrombosis sealing the rupture is low in such a short occlusion time. Thus, the reversal of the anticoagulant is crucial.

Unfractionated heparin, enoxaparin and bivalirudin are recommended by percutaneous coronary intervention guidelines for anticoagulation during the PCI [37]. The unfractionated heparin dose should be adjusted to ensure an activated clotting time (ACT) of 250–350 s during PCI; for patients using glycoprotein IIb/IIIa inhibitors, the ACT time should be maintained at 200–250 s. Meanwhile, an ACT of <150–180 s is recommended for artery sheath withdrawal. There is no clear recommendation regarding which level of the heparin anticoagulant effect should be maintained after CAP. Because intervention devices remain in the patient, the heparin effect should not be completely reversed, and it might be acceptable to maintain the ACT at 150–250 s. The ACT should be measured immediately after CAP. Protamine can be used, according to recommendations, and the ACT should be closely monitored. Further treatment is dependent on the bleeding level and hemodynamic status. Low-molecular-weight heparin can be partially reversed by protamine, and the dosage depends on the anti-Xa activity [38].

The bivalirudin anticoagulation effect linearly depends on its dosage, and the recommended monitoring parameter is the ACT; however, no antagonist exists for bivalirudin [39]. The half-life of bivalirudin is, theoretically, 25 min; when the effect of the drug is half reduced after one half-life, thrombosis is a possibility to achieve a successful sealing in a low-pressure environment. The risk of thrombosis must be considered after more than two half-lives. It is still controversial whether bivalirudin is safer than unfractionated heparin in CAP patients [40]. In guide wire-induced CAP, adverse cardiovascular events occurred more often in the unfractionated heparin group than in the bivalirudin group [41]. The incidence of pericardial tamponade was lower, but the mortality rate was higher in bivalirudin group [42].

## 4. Recommended Procedure

**Figure 1:**
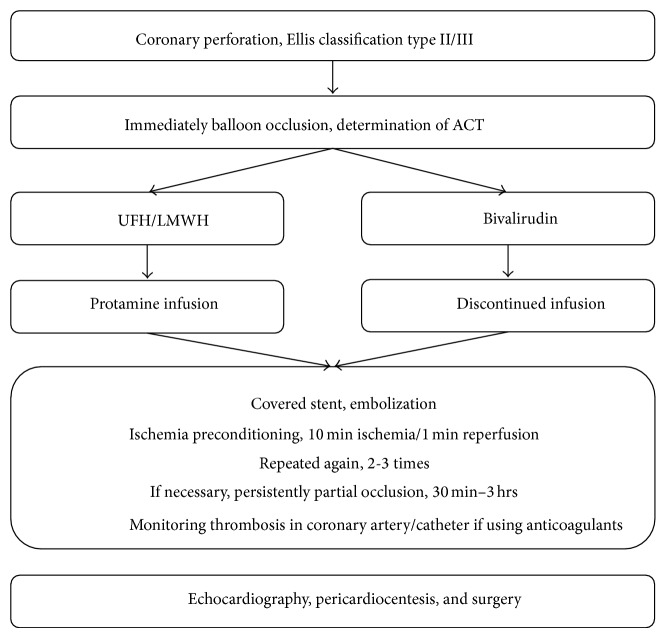
Schematic of recommended CAP treatment.

When CAP occurs, suspend anticoagulants and determine the ACT immediately Figure 1. A covered stent may be a lifesaving choice if it is available; however, its long-term effect is not satisfactory due to the high incidence of restenosis. Protamine can be administered to partially reverse the anticoagulation effect of unfractionated heparin or low-molecular-weight heparin according to the ACT.

As an adjunctive or alternative method, balloon occlusion can be used to treat the bleeding in CAP. Whatever balloon occlusion model is used, cardiac function inevitably decreases due to myocardial ischemia. Cardiogenic shock may occur in patients with marginally compensated chronic heart failure. Thus, it is important to rapidly evaluate the patient's basal cardiac function, the myocardial area at risk, and its tolerability to ischemia. IPC is the best choice and can be performed using 10 min ischemia/1 min reperfusion model and can be repeated again if necessary. If the bleeding is still not stopped, a persistent partial occlusion with the TIMI flow grade 2 or the distal MAP at approximate 40 mmHg can be performed for up to several hrs. Pericardiocentesis can also be performed to restore the local coronary perfusion status and systemic hemodynamics. If necessary, cardiac surgery should be performed within 2-3 hrs.

## 5. Conclusion

Although CAP is an uncommon complication of PCI, the acute and long-term prognosis of type III CAP is unsatisfactory. There are several reasons to believe that the balloon occlusion model can be optimized. We believe that preconditioning occlusion treatment should be applied first and repeatedly. If necessary, a partial occlusion treatment can be used as an alternative choice. This therapeutic method merits further investigation.
